# A Low-Noise Amplifier for Submarine Electric Field Signal Based on Chopping Amplification Technology

**DOI:** 10.3390/s24051417

**Published:** 2024-02-22

**Authors:** Fenghai Liu, Shaoheng Chun, Rujun Chen, Chao Xu, Xun Cao

**Affiliations:** 1 School of Geoscience and Info-Physics, Central South University, Changsha 410083, China; 215011090@csu.edu.cn (F.L.); 225012135@csu.edu.cn (C.X.); 215011093@csu.edu.cn (X.C.); 2 AIoT Innovation and Entrepreneurship Education Center for Geology and Geophysics, Central South University, Changsha 410083, China; chunsh123@126.com; 3 Control Technology Institute, Wuxi Institute of Technology, Wuxi 214121, China; 4 Key Laboratory of Metalorganic Prediction of Nonferrous Metals and Geological Environment Monitoring (Central South University), Ministry of Education, Changsha 410083, China; 5 Hunan Key Laboratory of Non-Ferrous Resources and Geological Hazard Detection, Changsha 410083, China

**Keywords:** low-noise amplifier, submarine electric field, chopping amplification technology, circuit simulation, noise measurement, equivalent input voltage noise spectrum (EIVNS)

## Abstract

In the exploration of ocean resources, the submarine electric field signal plays a crucial role through marine electromagnetic methods. However, due to the field signal’s low-frequency and weak characteristics, it often encounters interference from the instrument’s own 1/f noise during its acquisition. To address this issue, we developed a low-noise amplifier for the submarine electric field signal based on chopping amplification technology. This amplifier utilizes low-temperature electronic components to adapt to the cold submarine environment and enhances its independence by incorporating a square wave generator. Additionally, we conducted simulations and experimental tests on the designed chopper amplifier circuit, evaluating the equivalent input voltage noise spectrum (EIVNS) and the frequency response within 1 mHz~100 Hz. The experimental results indicate that the amplifier designed in this study achieves sufficiently low noise 2 nV/√Hz@1 mHz, effectively amplifying the submarine electric field signal measured with the electric field sensor.

## 1. Introduction

In recent years, the measurement of submarine electric fields has assumed increasing significance. In the domain of national defense, crucial applications such as electromagnetic stealth [1] and underwater target identification [2,3] are fundamentally reliant on the acquisition and utilization of electric field signals. Within the field of geophysics, the extensive exploitation of terrestrial mineral resources has yielded elevated production costs; therefore, the imperative of exploring and exploiting the abundant submarine mineral reservoirs has been underscored [4]. This becomes pivotal for ventures dedicated to the exploration of submarine hydrocarbon reserves [5], natural gas hydrates [6,7], and polymetallic sulfides [8]. Furthermore, electric field signals play an indispensable ancillary role in the comprehensive examination of extensive submarine geological structures, including mid-ocean ridges [9], submarine volcanoes, and subduction zones [10]. In the realm of physical oceanography, the electric field signal assumes a pivotal role in the elucidation of aqueous fluid dynamics [11].

Theory corroborates that high-frequency electromagnetic waves are largely ‘shielded’ by seawater, whereas low-frequency electromagnetic signals can effectively ‘penetrate’ the seawater and reach the ocean floor ([12], pp. 5–6). However, it is imperative to acknowledge that the strength of low-frequency electric field signals at the level of the seafloor is exceedingly feeble. To illustrate, in an illustrative scenario located 200 km southwest of San Diego, featuring a water depth of 3700 m, a 1 mHz electric field signal registered an amplitude with a mere magnitude of 10^−8^ V/m [13]. Signals of higher frequencies exhibited even more diminished amplitudes [14]. Notably, J.H. Filloux has elucidated the formidable challenges associated with the precise recording of submarine electric field signals, arising from the dual constraints of their diminutive amplitudes and the substantial and inescapable noise interference at the interface of seawater and measurement instrumentation [15].

Due to the low-frequency and weak characteristics of submarine electric field signals, obtaining accurate signals is quite challenging, necessitating the support of submarine electric field measurement that introduces low noise levels [16]. Sensitive electric field sensors are essential components of submarine electric field measurement. The low-noise amplifier used to amplify the low-frequency and weak signals output by the electric field sensor is even more crucial. Chopping amplification technology is the preferred technique for this low-noise amplifier due to its ability to avoid 1/f noise through spectrum migration [17].

In the past, some scholars have applied chopping amplification technology to the amplification of submarine electric fields. Constable et al. [18,19] designed a chopper amplifier with an EIVNS of approximately 2 nV/√Hz@1 mHz. However, the circuit required an external square wave signal, and the components LP311M and TL032ACD used in the design operated at temperatures above 0 degrees Celsius, which may not be well suited for the low-temperature environment of the seabed. Liu et al. [20] also developed a chopper amplifier, but its noise increased significantly below 0.01 Hz, making it challenging for the amplifier to meet the low-noise requirements. Chen et al. [21] designed a similar amplifier that featured low noise at 3 nV/√Hz@1 mHz, but the circuit also required an external square wave signal. There remains a lack of clarity as to whether Chen’s design is able to meet the low-temperature operating conditions of the seabed.

In this study, on the basis of previous research, a chopper amplifier with a self-contained square wave generator was designed, more suited to the low-temperature environment of the seabed. Firstly, the chopper amplifier was divided into seven modules, and each module was investigated in detail. Simultaneously, the fundamental principles of chopping amplification technology were analyzed within the frequency domain. Subsequently, using Multisim software 14, this study simulated the designed chopper amplifier circuit, tested the input and output waveforms, obtained the frequency response, and estimated the simulated equivalent EIVNS through a combined approach with noise theory. This validated the feasibility of the circuit. Following that, this study conducted tests on the corresponding physical circuit, obtaining measured EIVNS and frequency responses, while providing a detailed explanation of the EIVNS measurement method. Furthermore, this study undertook a thorough analysis of both the simulated and experimental results. Lastly, the advantages of the amplifier were extracted, and its significance in submarine electric field measurement was summarized.

## 2. Chopper Amplifier Circuit Principle

This study employed chopping amplification technology to modulate the low-frequency 1 mHz–100 Hz [22] electric field signal into the high-frequency range of 2 kHz. Subsequently, the modulated signal was input into an operational amplifier for amplification. This approach effectively mitigates a substantial portion of the 1/f noise inherent in operational amplifiers. According to the chip manual of MAX4101, the corner frequency of voltage noise for this chip was approximately 2 kHz, and the voltage noise was approximately 7 nV/√Hz@2kHz. When the signal frequency was modulated to 2 kHz, the signal remained unaffected by the low-frequency 1/f noise. After a certain degree of amplification, the signal was demodulated back to its original low-frequency range of 1 mHz–100 Hz. The slight offset and noise introduced by the operational amplifier were only subject to the demodulation process, shifting the spectrum to higher frequencies of 2 kHz. Finally, after passing through a low-pass filter, the high-frequency offset and noise were filtered out, retaining the amplified low-frequency signal. This ultimately accomplished the amplification of weak, low-frequency signals [23].

**Figure 1:**
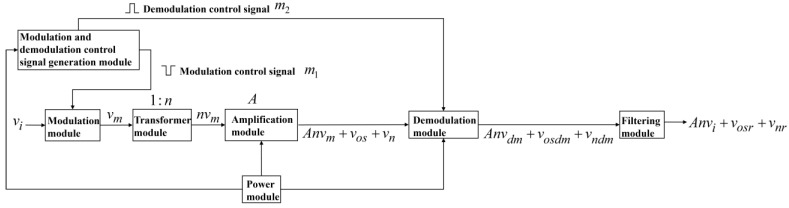
Schematic diagram of the operational principles of the chopper amplifier circuit.

The chopper amplifier circuit designed in this paper can be divided into seven modules, as follows: the modulation and demodulation control signal generation module, the modulation module, the transformer module, the amplification module, the demodulation module, the filtering module, and the power module. The schematic diagram of its operational principles is depicted in Figure 1.

The main function of the modulation and demodulation control signal generation module is to generate the control square wave signals required for modulation and demodulation. The module comprises a square wave generator and an inverter. The square wave generator is responsible for generating square waves, while the inverter introduces a 180-degree phase shift to the generated square wave, thereby producing a set of square waves with opposite phases ($m_{1}$ and $m_{2}$ in Figure 1), used for modulation and demodulation control, respectively. This set of square wave signals exhibits equal amplitude $A_{1}$ and frequency $f_{chop}$, with opposite phases. $f_{chop}=2\;kHz$ in this paper.

**Figure 2:**
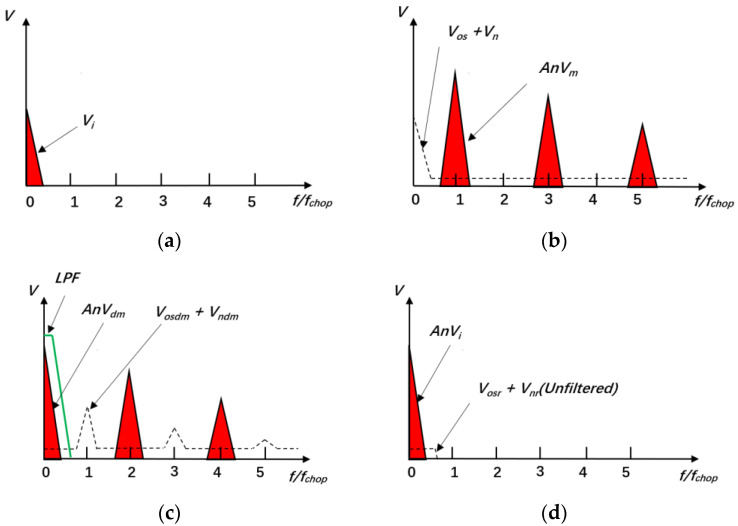
Spectrum variation diagram of chopper amplifier circuit signal. (**a**) The spectrum of $v_{i}$; (**b**) the spectrum of the signal passing through amplification module; (**c**) the spectrum of the signal passing through demodulation module; (**d**) the spectrum of the signal passing through filtering module.

The modulation module modulates the voltage signal $v_{i}$, from the electric field sensor, into the signal $v_{m}$ using modulation control signal $m_{1}$ in Figure 1. In Figure 2a, the spectrum of $v_{i}$ is depicted. $V_{m}(w)$, $V_{i}(w)$, and $M_{1}(w)$ are the Fourier forms of $v_{m}\left(t\right)$, $v_{i}\left(t\right)$, and $m_{1}\left(t\right)$ in Equation (1), respectively. Modulation induces a spectral shift in the signal, and this process is expressed in the frequency domain as follows:

(1) $$V_{m}(w)=\frac{1}{2\pi }V_{i}(w)*M_{1}(w)$$

where $M_{1}(w)=-4A_{1}[\delta (w-w_{1})+\frac{1}{3}\delta (w-3w_{1})+\frac{1}{5}\delta (w-5w_{1})+...+\frac{1}{k}\delta (w-kw_{1})]i$; $w_{1}=2\pi f_{chop};$ and k=1, 3, 5,… (*k* is an odd number). Based on the properties of the im-pulse function, when $w=kw_{1}$, the value of $M_{1}(w)$ is not equal to zero, but when $w\neq kw_{1}$, the value of $M_{1}(w)$ equals zero. According to the convolution property, at this point, the value of $V_{m}(w)$ is consistent with $M_{1}(w)$ in Equation (1). In other words, following modulation, the energy distribution of the resultant signal, $v_{m}$, primarily resides within the odd harmonics of the modulating signals, $m_{1}$ or $m_{2}$, as depicted in Figure 2b [23].

The transformer module amplifies the modulated signal $v_{m}$ into the signal $nv_{m}$, as illustrated in Figure 1. The primary functions of the transformer module encompass noise matching, signal amplification, and isolation. By utilizing a transformer with a turn ratio of 1:n, the transformer module amplifies the modulated signal $v_{m}$ by a factor of n while increasing the electrode’s impedance by $n^{2}$ times, aiming to achieve optimal source impedance matching [24] with the primary amplification circuit, and thereby reducing noise. The objective of isolation is to ensure that there is no impedance connection between the instrument’s ‘ground’ and the grounded electrode [18].

The amplification module magnifies the signal $nv_{m}$ into a signal represented as $Anv_{m}+v_{os}+v_{n}$, as depicted in Figure 1. The amplification module comprises both a primary amplification circuit and a secondary amplification circuit, yielding an overall gain which is denoted as A. The rationale for utilizing a two-stage amplification circuit lies in the fact that, despite the initial amplification via the transformer, the signal $nv_{m}$ remains relatively weak. The primary amplification circuit predominantly consists of discrete amplifying transistors, possessing lower-noise characteristics than operational amplifiers. This architecture enables the signal to undergo further amplification before transmission to the operational amplifier in the secondary amplification circuit, thus mitigating the risk of the operational amplifier’s noise overwhelming the signal ([25], pp. 79–80).

Considering that the signal $nv_{m}$ experiences a spectral shift, transitioning from a lower frequency to a higher frequency, this approach mitigates the introduction of significant 1/f noise attributable to the operational amplifier. Nevertheless, it does introduce a certain offset $v_{os}$ and noise $v_{n}$, encompassing shot noise, thermal noise, and a minor amount of 1/f noise, as illustrated in Figure 2b.

The demodulation module, as depicted in Figure 1, employs a demodulation control signal to demodulate the signal $Anv_{m}+v_{os}+v_{n}$ into $Anv_{dm}+v_{osdm}+v_{ndm}$. $V_{dm}(w)$, $V_{i}(w)$, and $M_{1,2}(w)$ are the Fourier forms of $v_{dm}\left(t\right)$, $v_{i}\left(t\right)$, and $m_{1}\left(t\right)m_{2}\left(t\right)$ in Equation (2), respectively. This demodulation process also involves a spectral shift, which can be represented in the frequency domain as follows:

(2) $$V_{dm}\left(w\right)=\frac{1}{2\pi }V_{i}\left(w\right)*M_{1,2}(w)$$

where $M_{1,2}\left(w\right)=-\frac{16{A_{1}}^{2}}{\pi^{2}}\left[a_{0}\delta \left(w\right)+a_{1}\delta \left(w-2w_{1}\right)+\ldots +a_{k}\delta \left(w-2nw_{1}\right)\right]$; $w_{1}=2\pi f_{chop};$ n=0, 1, 2,…(natural numbers); and $a_{0},\;a_{1},...,a_{n}\neq 0$. Based on the properties of the impulse function, when $w=2nw_{1}$, the value of $M_{1,2}\left(w\right)$ is not equal to zero, but when $w\neq 2nw_{1}$, the value of $M_{1,2}\left(w\right)$ equals zero. According to the convolution property, at this point, the value of $V_{dm}\left(w\right)$ is consistent with $M_{1,2}\left(w\right)$ in Equation (2). In other words, following modulation, the energy distribution of the resultant signal, $v_{dm}$, primarily resides within the even harmonics of the modulating signals, $m_{1}$ or $m_{2}$, as depicted in Figure 2c. The spectral migration process of offset voltage $v_{os}$ and noise $v_{n}$ during demodulation mirrors the spectral migration process of the modulated signal $v_{i}$, indicating that the post-demodulation offset, $v_{osdm}$, and noise, $v_{ndm}$, predominantly distribute their energy within the odd harmonics of the control signals, $m_{1}$ or $m_{2}$, as illustrated in Figure 2c [23].

The filtering module processes the demodulated signal $Anv_{dm}+v_{osdm}+v_{ndm}$ to obtain the signal $Anv_{i}+v_{osr}+v_{nr}$, as illustrated in Figure 1. Based on the spectral components of the post-demodulation signal, it can be inferred that the effective signal, $Anv_{dm}$, is demodulated back to its original frequency range, situated in the low-frequency domain, while the offset, $v_{osdm}$, and noise, $v_{ndm}$, remain demodulated and occupy the high-frequency domain. Consequently, the filtering module employs a low-pass filter (LPF) to eliminate a significant portion of the high-frequency noise $v_{ndm}$ and offset $v_{osdm}$, preserving the low-frequency signal, ${Anv}_{i}$, along with a minor fraction of the offset $v_{osr}$ and noise $v_{nr}$. The bandwidth of this LPF [26] is depicted in Figure 2c. Figure 2c,d illustrate the spectral changes before and after signal filtering. Ultimately, the low-frequency and weak signals from the electric field sensor are subject to low-noise amplification following their passage through the filtering module.

## 3. Simulation of the Chopper Amplifier Circuit

**Figure 3:**
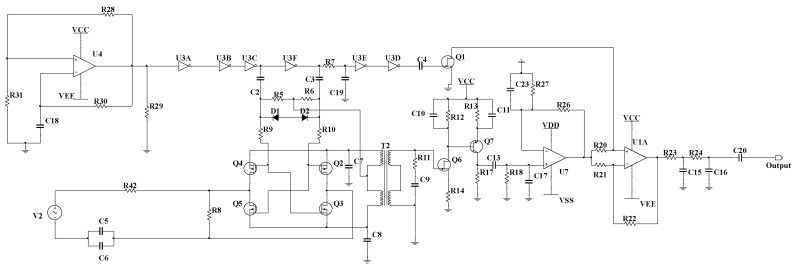
Simulation circuit diagram of chopper amplifier circuit.

We designed a chopper amplifier circuit within the Multisim software [27], as depicted in Figure 3. The circuit designed in this study placed strong emphasis on the low-temperature working environment of the seabed, and Table 1 presents the operating temperatures of the electronic components used in Figure 3. In addition, the modulation and demodulation control signal generation module mentioned in Figure 1 consisted of U4, R31, R28, R30, R29, and C18 in Figure 3. The design of this module will make the entire circuit work more independently without the need to introduce an additional signal source from outside.

Within the Multisim environment, considerations for resistors are primarily confined to thermal noise, while semiconductors are accounted for in terms of thermal noise, shot noise, and 1/f noise [27]. The primary objectives of this simulation encompassed two aspects: firstly, employing a specific sinusoidal wave as the test signal to observe its output waveform ([28], pp. 75–76), and secondly, estimation of its theoretical EIVNS $e_{simulated}$.

### 3.1. Gain Measurement

**Figure 4:**
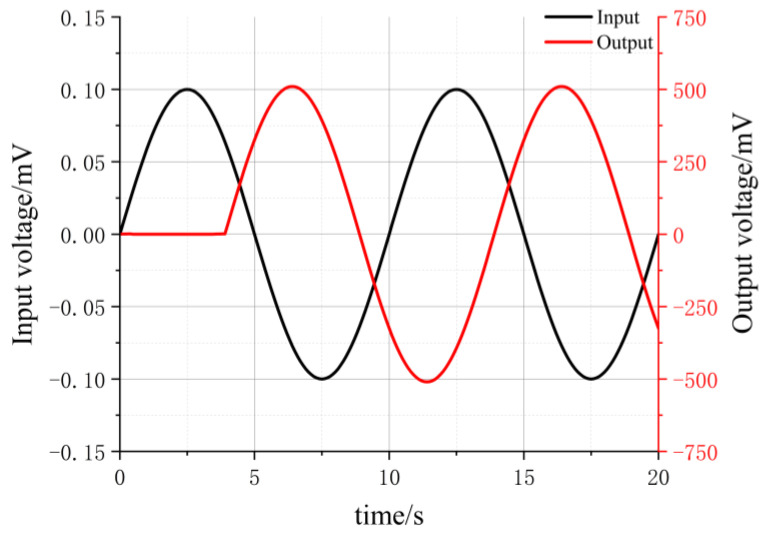
Comparison chart of simulated input and output sine waves.

In Figure 3, the signal source V2 simulates the input voltage signal from the electrode. V2 is set to a peak value of 0.1 mV and a frequency of 0.1 Hz for simulating a sine wave voltage signal. The output voltage is measured at the output terminal. The input voltage waveform is represented by the black curve, and the output voltage waveform is represented by the red line in Figure 4. The near-zero amplitude of the output voltage waveform at 0–4 s is due to the simulation transient response delay. Figure 4 indicates that the amplitude of the output voltage sine wave is approximately 506 mV, implying a gain of approximately 5060.

**Figure 5:**
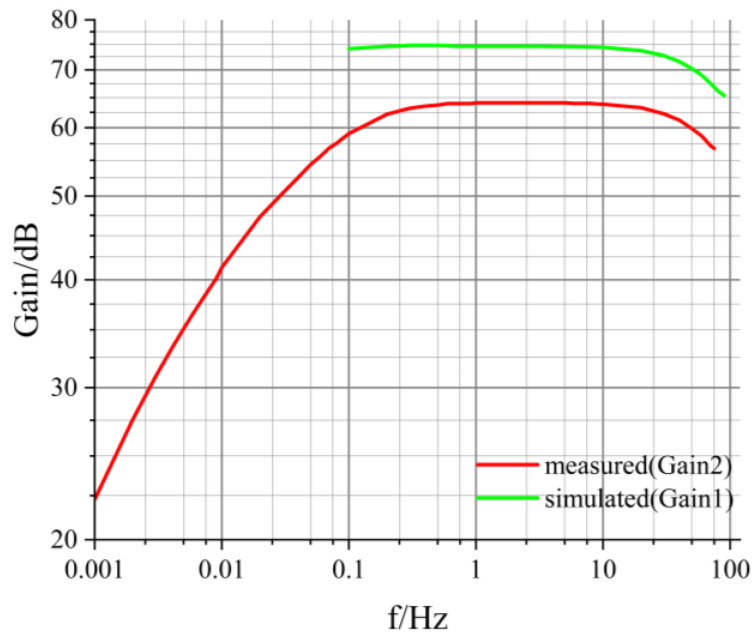
Simulated and measured frequency response of the chopper amplifier circuit.

The simulated frequency response of the amplifier is depicted as a cyan curve in Figure 5. However, due to limitations in storage space for simulation data within the Multisim software, and the increasing memory demands for lower-frequency simulated signals, the software may encounter memory limitations and thus cannot simulate frequencies lower than 0.1 Hz. Consequently, the cyan curve in Figure 5 only displays the gain simulation results for the frequency range from 0.1 Hz to 100 Hz.

### 3.2. Estimation of Theoretical Equivalent Input Noise Spectral Density

The circuit employs chopping amplification technology, leading to a spectral shift in the signal [17]. However, the Multisim software cannot directly measure the noise variation resulting from this spectral shift. To estimate the correct $e_{simulated}$ for the chopper amplifier circuit, this study utilized a combined approach of noise theory estimation and Multisim software measurements. Specifically, it involved aggregating the noise voltage generated from five different sections and equating it to the input, resulting in the derived value of $e_{simulated}$.

**Figure 6:**
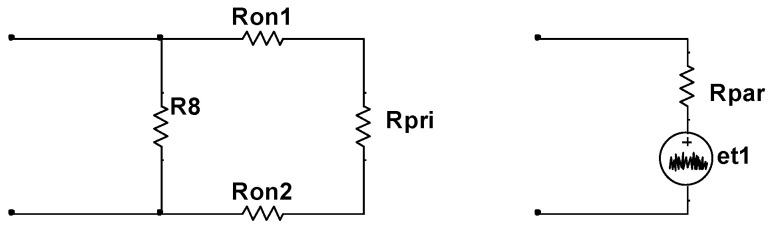
The equivalent model for the noise voltage in the first section.

The noise voltage in the first section is generated by a resistor $R_{8}$; MOSFETs $Q_{2}$, $Q_{3}$, $Q_{4}$, and $Q_{5}$; and the primary coil of transformer T2, as depicted in Figure 3. The resistance value of $R_{8}$ is 5.62 kΩ, the ON-state resistance of the MOSFETs ($R_{on}$) is approximately 5 Ω, and the equivalent resistance of the primary coil of T2 ($R_{pri}$) is approximately 7.5 Ω. The equivalent circuit for the first section is illustrated in the diagram on the left of Figure 6, and its thermal noise model is presented in the diagram on the right of Figure 6. In Figure 6, $R_{par}=(R_{on1}+R_{on2}+R_{pri})//R_{8}$, corresponding to the RMS thermal noise voltage, denoted as $E_{par}=\sqrt{4\;kTR_{par}B_{w}}$, where $B_{w}=(f_{up}-f_{down})w$, with $f_{up}=40\;Hz$ and $f_{down}<0.1\;Hz$, is the noise bandwidth, as $f_{up}\gg f_{down}$. Therefore, $B_{w}\approx f_{up}w=62.8\;Hz$, where w=1.57 is the noise bandwidth factor [20]. At room temperature, $4\;kT\approx 1.6\times 10^{-20}V^{2}/(Hz\cdot Ω)$ ([25], pp. 42). The values for $B_{w}$ and 4 kT remain consistent throughout.

The noise voltage in the second section is generated by the secondary coil of transformer T2, as depicted in Figure 3. The equivalent resistance of the T2 secondary coil, denoted as $R_{sec}$, is approximately 300 Ω, resulting in the RMS thermal noise voltage denoted as $E_{sec}=\sqrt{4kTR_{sec}B_{w}}$.

**Figure 7:**
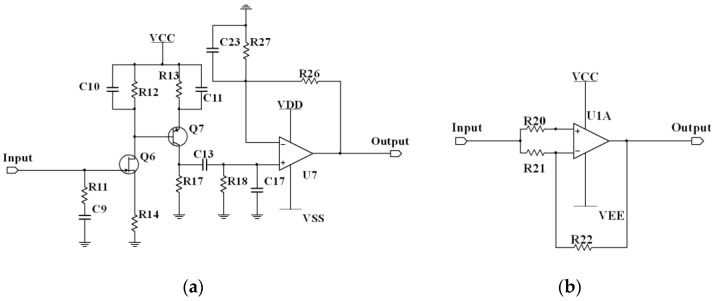
(**a**) Schematic diagram of the amplification module. (**b**) Schematic diagram of the demodulation module.

The noise voltage in the third section is generated by the amplification module of the chopper amplifier circuit, as illustrated in Figure 7a. Given the intricate nature of the noise contributions in this section, to achieve a more accurate estimation of the noise voltage produced, this study employed Multisim software for noise measurements. The obtained EIVNS $e_{NA}$ ≈ 1.20 nV/√Hz@2 kHz, which corresponds to the RMS denoted as $E_{A}=e_{NA}\sqrt{B_{w}}$.

The noise voltage in the fourth section is generated by the demodulation module of the chopper amplifier circuit, as shown in Figure 7b. In the Multisim software, the EIVNS $e_{ND}$ of the demodulation module is approximately 83.02 $\frac{nV}{\sqrt{Hz}}@1\;mHz-10\;kHz$, corresponding to the RMS denoted as $E_{D}=e_{ND}\sqrt{B_{w}}$.

The noise voltage in the fifth section is generated by the series combination of resistors $R_{23}=11.3\;kΩ$ and $R_{24}=249\;kΩ$ within the filter module of the chopper amplifier circuit, as depicted in Figure 3. The RMS thermal noise voltage generated by $R_{23}$ and $R_{24}$ in series is denoted as $E_{ser}=\sqrt{{4\;kT(R_{23}+R_{24})B}_{w}}$.

Based on the above analysis, Table 2 presents the calculated values of the five sections of noise and their corresponding magnification at the input end.

**Figure 8:**
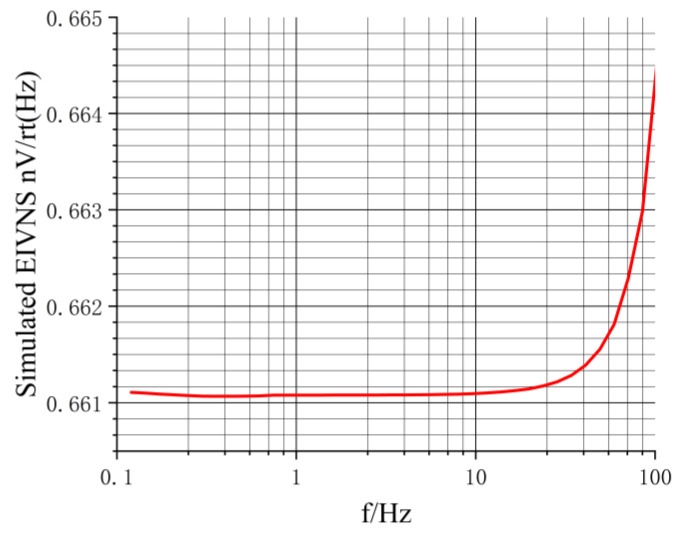
The simulated EIVNS.

The formula for calculating the noise voltage, equivalent to the input, originating from five distinct sections, is as follows:

(3) $$e_{simulated}=\sqrt{{E_{par}}^{2}+{\left(\frac{E_{sec}}{n}\right)}^{2}+{\left(\frac{E_{A}}{n}\right)}^{2}+{\left(\frac{E_{D}}{Gain1}\right)}^{2}+{\left(\frac{E_{ser}}{Gain1}\right)}^{2}}/\sqrt{B_{w}}$$

where n represents the gain of transformer $T_{2}$, as depicted in Figure 3, and Gain1 signifies the simulated gain as shown in Figure 5. Note: during calculation, Gain1 should be converted to the corresponding magnification. The value of $e_{simulated}$ is obtained from Equation (3), as illustrated in Figure 8.

In Figure 8, $e_{simulated}$ increases with frequency. As the frequency increases, Gain1 in Equation (3) decreases, as shown in Figure 5, and $e_{simulated}$ is inversely proportional to Gain1, so $e_{simulated}$ increases. However, the overall range remains within 0.661–0.665 nV/√Hz, reaching a noise level within the reasonable range [18,19,20,21,29,30].

The simulated results validate the feasibility of the chopper amplifier circuit designed in this study, providing theoretical support for the fabrication of an actual circuit.

## 4. Measurement of the Chopper Amplifier Circuit

**Figure 9:**
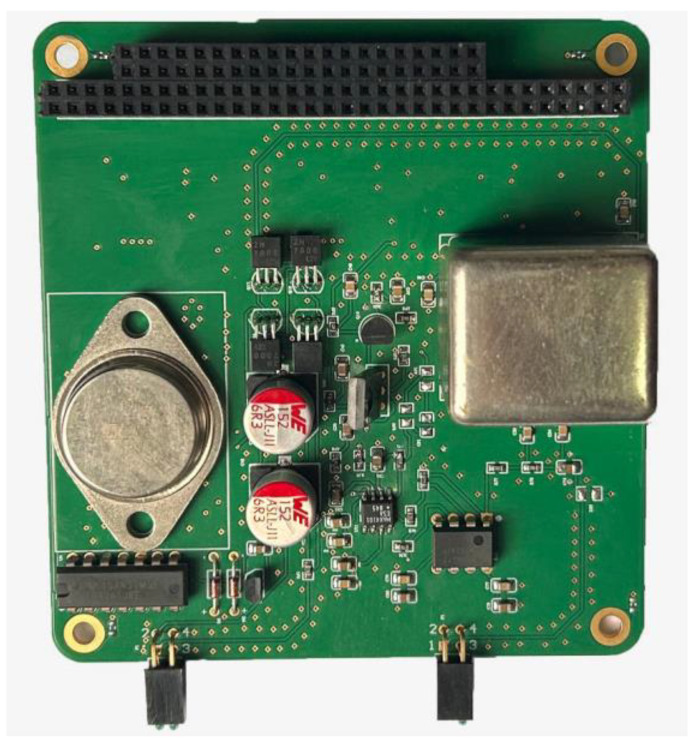
The chopper amplifier circuit’s PCB.

Based on the simulation results of the chopper amplifier circuit in Multisim, we utilized Altium Designer version 21.0.8 to create the corresponding schematic diagram and PCB layout. Subsequently, the PCB was fabricated, as depicted in Figure 9.

### 4.1. Gain Measurement

**Figure 10:**
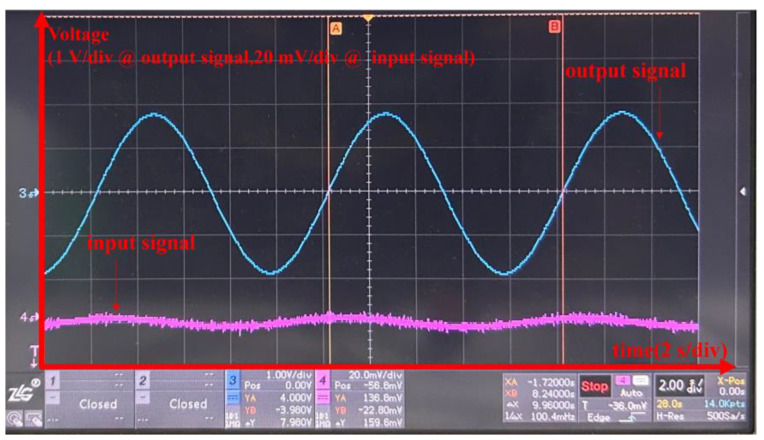
A comparative diagram of input signal and output signal.

We employed a sinusoidal voltage signal with an amplitude of 2 mV and a frequency of 0.1 Hz as the input signal. Simultaneously, we used an oscilloscope to measure the input and output of the circuit board. The results are indicated in Figure 10. In Figure 10, it can be observed that the amplitude of the output waveform is approximately 1.8 V, indicating a gain of approximately 900 times at 0.1 Hz. A and B in Figure 10 are auxiliary lines for confirming waveforms’ cycle.

The measured frequency response of the amplifier is illustrated by the red curve in Figure 5. As is evident from Figure 5, the passband gain of the circuit board is approximately 64 dB, with a −3 dB bandwidth spanning from approximately 0.15 Hz to 40 Hz.

When comparing the gain obtained from the simulation of the chopper amplifier circuit with the measured magnification (refer to Figure 5), it is evident that the measured values are significantly lower. There are two potential reasons for this observation. Firstly, real-world components may exhibit non-ideal characteristics, including internal losses in components such as resistors, capacitors, and inductors, which can lead to a reduction in the measured circuit’s gain. Secondly, variations in component parameters due to manufacturing differences may cause the component values to deviate from their nominal specifications. For example, the measured AC gain of a transistor may be lower than its nominal value, resulting in a decrease in the circuit’s gain.

### 4.2. Measurement of EIVNS

In order to quantify the noise level of the circuit board, it is necessary to obtain the EIVNS $e_{measured}$ of the board [31]. To acquire $e_{measured}$, $e_{C+I}$ and $e_{I}$ must be measured. $e_{C+I}$: the RMS spectrum of the output noise voltage generated by the combined action of the chopper amplifier board and the voltage measurement instrument [32,33]. $e_{I}$: the RMS spectrum of the output noise voltage generated by the voltage measurement instrument alone. The relationship between them can be expressed as follows:

(4) $$e_{measured}=\frac{\sqrt{e_{C+I}^{2}-e_{I}^{2}}}{A^{\prime }}$$

where $A^{\prime }=32Gain2$, where Gain2 is as illustrated in Figure 5 and 32 represents the gain of the voltage measurement instrument [34]. Note: during calculation, Gain2 should be converted to the corresponding magnification. The purpose of Equation (4) is to eliminate the noise impact brought in by the voltage measurement instrument during the noise measurement process and to be equivalent to the noise at the input end.

**Figure 11:**
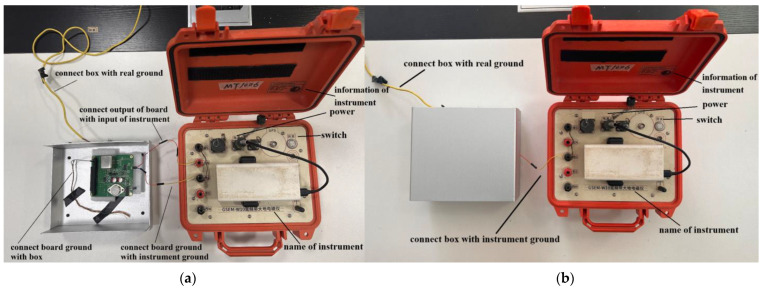
Two configurations of noise measurement for $e_{C+I}$ and $e_{I}$. (**a**) The configuration for measuring $e_{C+I}$; (**b**) the configuration for measuring $e_{I}$.

To obtain the $e_{C+I}$, we shorted the input of the circuit board. Subsequently, we placed the board within a shielded enclosure to mitigate external electromagnetic interference ([25], pp. 133–134). In addition, we interconnected the grounding of the circuit board, the shielded enclosure, and the voltage measurement instrument with the actual ground. Following this, we used a wire to connect the output of the board to the input of the voltage measurement instrument located outside the shielded enclosure. Additionally, the voltage measurement instrument was set to a 32-fold amplification mode. Figure 11a illustrates this measurement configuration.

**Figure 12:**
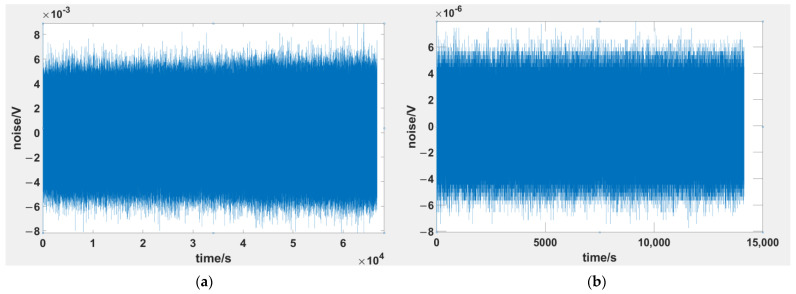
The voltage time series for the two configurations. (**a**) The voltage time series of $e_{C+I}$; (**b**) the voltage time series of $e_{I}$.

**Figure 13:**
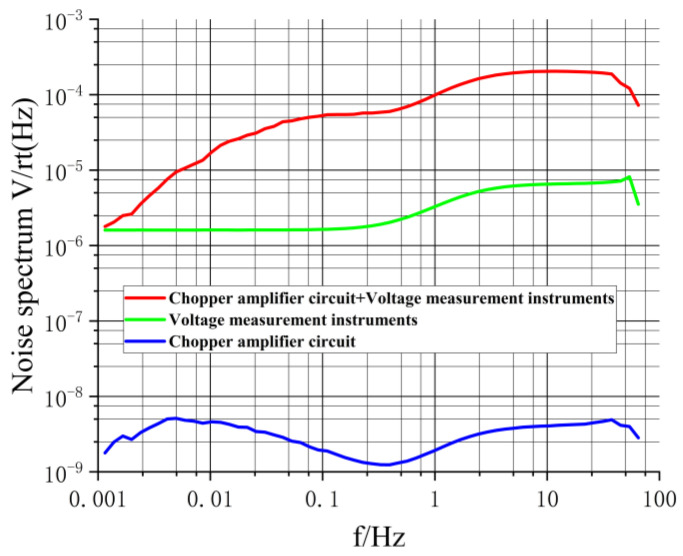
The spectrum of noise voltage for the chopper amplifier circuit/voltage measurement instrument.

Following the measurement configuration shown in Figure 11a, the voltage measurement instrument continuously collected data for approximately 69,000 s, with a sampling rate of 150 Hz [35], resulting in a time series of noise voltage as depicted in Figure 12a. The peak-to-peak value of this noise was approximately 12 mV. A Fourier transformation was applied to the time series of the noise voltage, along with specific data processing, to obtain the red curve in Figure 13, denoted as $e_{C+I}$.

To measure $e_{I}$, we shorted the input of the voltage measurement instrument. We connected the grounding of the voltage measurement instrument and the shielded enclosure to the actual ground, and the voltage measurement instrument was set to a 32-fold amplification mode. Figure 11b illustrates this measurement configuration. Following this setup, the voltage measurement instrument continuously collected data for approximately 14,000 s, with a sampling rate of 150 Hz, resulting in a time series of noise voltage as shown in Figure 12b. The peak-to-peak value of this noise was approximately 12 μV. A Fourier transformation was applied to the time series of the noise voltage, along with specific data processing, to obtain the cyan curve in Figure 13, denoted as $e_{I}$.

In accordance with Equation (4), $e_{measured}$ is obtained, as depicted by the blue curve in Figure 13. In general, $e_{measured}$ remains at a noise level on the order of nV/√Hz, meeting the low-noise requirements for amplifying submarine electric field signals [27,29,30].

## 5. Discussion and Analysis

**Figure 14:**
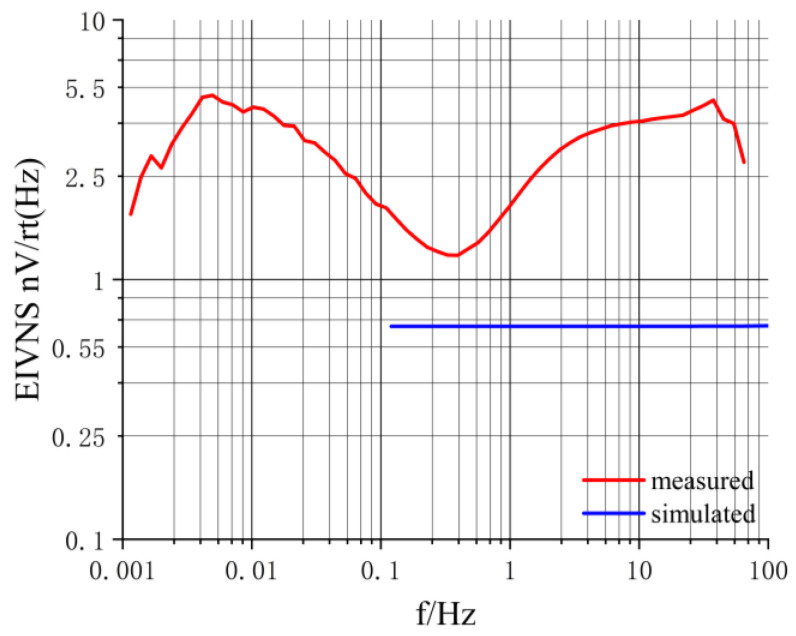
EIVNS of the chopper amplifier circuit in simulation and experimentation.

We combined simulation with experimentation to investigate the chopper amplifier circuit. The simulation provides theoretical support for the actual circuit design, reduces the development costs, and shortens the development cycle. Figure 14 displays the EIVNS of the circuit in both simulated ($e_{simulated}$) and experimental ($e_{measured}$) scenarios. $e_{simulated}$ is approximately 0.66 nV/√Hz. $e_{measured}$ exhibits a maximum value of approximately 5.00 nV/√Hz@0.005 Hz and 40 Hz and a minimum value of around 1.20 nV/√Hz@0.4 Hz. That means that $e_{measured}$ is approximately 1.8 to 7.6 times greater than $e_{simulated}$.

We determined that there might be three reasons for the discrepancies between the simulation and actual results. First, the physical layout and wiring of the circuit board can influence its intrinsic noise. In the simulation, components can be ideally arranged, while in the actual circuit, the layout and wiring are not as perfect as in the simulation. Second, the voltage measurement instrument used for testing the chopper amplifier circuit board can introduce errors, with the instrument’s accuracy and resolution impacting the noise measurement results. Third, factors such as temperature, humidity, and other conditions affect the noise performance of electronic components. Simulation software like Multisim typically does not account for these factors, but they can influence the actual circuit. For example, MOSFETs in the modulation module inject charges, which is not considered during simulations but does occur in reality, causing additional noise [36].

Table 3 presents the EIVNS of chopper amplifier circuits that are used as preamplifiers for detecting submarine electric field signals. The table represents the amplifiers designed in this study, at the Scripps Institution of Oceanography (SIO) in the United States [18], the Ocean University of China (OUC) [20], and the China University of Geosciences (CUG) [21].

In Table 3, the EIVNS of the circuit produced in this study, while generally close to those of other institutions (except for OUC’s frequencies of 0.001 Hz and 0.01 Hz), are not entirely identical. This is a normal occurrence because each institution employs different components, circuit layouts, wiring, and testing conditions and experiences varying degrees of external electromagnetic interference. This substantiates the reliability of the noise parameters of the circuit and further validates the feasibility of this design. Table 4 shows a comparison between this circuit and previous works [18,20,21]. This circuit not only has excellent performance in terms of noise, but also has certain advantages in low-temperature situations and independent operation.

## 6. Conclusions

In general, this study innovatively adopted a combination of noise theory estimation and Multisim software simulation to study the chopper amplifier circuit during the circuit design. The noise theory estimation method compensates for the distortion in noise estimation in the software simulation under spectrum shifting conditions, and simulation tests provide theoretical support for the practical circuit design. Based on this approach, a low-noise amplifier for submarine electric field signals based on chopping amplification technology was developed in this study. Both simulation and experimental results confirm the feasibility of the proposed design, and comparative results with peers demonstrate that the amplifier has the advantages of low noise, cold resistance, and standalone capacity. All these conclusions indicate that the amplifier developed in this study can effectively amplify submarine electric field signals measured using electric field sensors under low-temperature conditions, making it more suitable for the complex collection scenarios that exist in submarine exploration.

## Figures and Tables

**Table 1:** Temperature characteristics of main electronic components in chopper amplifier circuit.

Electronic Components	Operation Temperature
U4(AD3554SM)	−25–85 °C
U3(CD4069UBE)	−55–125 °C
Q2Q3Q4Q5(2N7000)	−55–150 °C
Q6(2SK170)	−55–125 °C
Q7(2N4918G)	−65–150 °C
U7(MAX4101ESA)	−40–85 °C
Q1(2SK330)	−55–125 °C
U1A(TL032AIP)	−40–85 °C

**Table 2:** Voltage noise analysis results for 5 sections.

RMS of Voltage	Source	Magnification	Value/nV
$E_{par}$	Modulation	1	4.1879
$E_{sec}$	Transformer	n	17.3660
$E_{A}$	Amplification	n	9.5215
$E_{D}$	Demodulation	Gain1	657.8652
$E_{ser}$	Filter	Gain1	606.9000

**Table 3:** The noise of chopper amplifier circuits from different institutions.

EIVNS/(nV/√Hz)				
Frequency/Hz	Ours	SIO	OUC	CUG
0.001	2.0	2.1	100.0	3.0
0.01	4.5	2.1	80.0	2.5
0.1	1.9	2.1	6.0	0.8
1	2.0	2.0	3.0	0.5
10	4.0	2.0	2.0	0.5

**Table 4:** A comparison of amplifier functionality with published work.

Functionality	Ours	SIO	OUC	CUG
Low noise	√	√	×	√
Cold resistance	√	×	\	\
Standalone	√	×	×	×

## Data Availability

Data are contained within the article.

## Abbreviation

Simple description for symbols		
**Mnemonic**	**Description**	**Unit**
EIVNS	equivalent input voltage noise spectrum	nV/√Hz
$v_{i}$	input signal of chopper amplifier circuit	V
$v_{m}$	signal modulated	V
n	magnification of transformer	\
A	magnification of amplification module	\
$m_{1}$	signal used to modulate	V
$m_{2}$	signal used to demodulate	V
$v_{os}$	offset generated from amplification module	V
$v_{n}$	noise generated from amplification module	V
$v_{dm}$	signal demodulated	V
$v_{osdm}$	$v_{os}$ demodulated	V
$v_{ndm}$	$v_{n}$ demodulated	V
$v_{osr}$	$v_{osdm}$ filtered	V
$v_{nr}$	$v_{ndm}$ filtered	V
$f_{chop}$	frequency of $m_{1}$ or $m_{2}$	kHz
$e_{simulated}$	estimation value of equivalent input noise spectral density	nV/√Hz
$e_{measured}$	experimental value of equivalent input noise spectral density	nV/√Hz
V2	source of input signal in Multisim	V
$B_{w}$	noise bandwidth	\
k	Boltzmann constant	J/K
T	temperature	K
$E_{par}$	RMS of noise voltage from modulation	nV
$E_{sec}$	RMS of noise voltage from transformer	nV
$E_{A}$	RMS of noise voltage from amplification	nV
$E_{D}$	RMS of noise voltage from demodulation	nV
$E_{ser}$	RMS of noise voltage from filter	nV
Gain1	magnification of chopper amplifier in simulation	\
Gain2	magnification of chopper amplifier in experimentation	\
